# Immunomodulatory effects of the polysaccharide from *Sinonovacula constricta* on RAW264.7 macrophage cells

**DOI:** 10.1002/fsn3.2735

**Published:** 2022-01-25

**Authors:** Zhidong Liu, Zhifang Liu, Laihao Li, Junjie Zhang, Qiancheng Zhao, Na Lin, Wenzhu Zhong, Mei Jiang

**Affiliations:** ^1^ East China Sea Fishery Research Institute Chinese Academy of Fishery Sciences Shanghai China; ^2^ South China Sea Fishery Research Institute Chinese Academy of Fishery Sciences Guangzhou China; ^3^ College of Food Science and Engineering Jiangsu Ocean University Lianyungang China; ^4^ College of Food Science and Engineering Dalian Ocean University Dalian China; ^5^ Fishery Machinery and Instrument Research Institute Chinese Academy of Fishery Sciences Shanghai China

**Keywords:** immunomodulatory activity, macrophage, polysaccharide, *Sinonovacula constricta*

## Abstract

This study aimed to evaluate the immunomodulatory effect of the polysaccharide from *Sinonovacula constricta* (SCP‐1‐1) in RAW264.7 cells. SCP‐1‐1 with a molecular weight of 440.0 kDa consisted of glucose and mannose. The immunomodulatory assay results showed that SCP‐1‐1 could significantly enhance phagocytic ability, NO production, and some cytokines (TNF‐α, IL‐6, and IL‐1β) secretion of RAW264.7 cell in a dose‐dependent manner. Western blot analysis results demonstrated that SCP‐1‐1 could regulate the expression levels of the key proteins in the signaling pathways of RAW264.7 cell and might associated with NF‐κβ and PI3K signaling pathway. These findings could contribute to elucidate the immunomodulatory activities of the polysaccharide from *Sinonovacula constricta*.

## Graphical abstract

The immunomodulatory activities of a novel polysaccharide (SCP‐1‐1) from *Sinonovacula constricta* were examined in RAW264.7 cells. These findings could contribute to elucidate the immunomodulatory activities of the polysaccharide from *Sinonovacula constricta*.

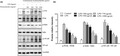

## 1 INTRODUCTION

*Sinonovacula constricta* is widely distributed in the intertidal zones and estuarine areas throughout the coast of the Western Pacific Ocean. *Sinonovacula constricta* has been cultured and used as food and traditional medicine for centuries in southeastern China due to the short culture cycle, unique flavor, good taste, and high nutritional value, which has been considered as one of the four most important and traditionally cultivated shellfish in China (Niu et al., 2014; Ran et al., 2017). In 2019, the production of cultured *S. constricta* was approximately 869,000 tons in China (2020). Recently, researchers have realized the healthy effect of *S. constricta* to be likely related to some bioactive components, such as polysaccharides and proteins (Wang et al., 2018).

Polysaccharides are biological macromolecules playing an important physiological role in plants, animals, and microorganisms (Xie et al., 2016). Polysaccharides from natural sources have showed excellent pharmacological effect, including anti‐diabetic, anti‐atherogenic, antitumor, antiviral, and immunomodulation activities (Ferreira et al., 2015; Martins et al., 2017; Liu et al., 2020). Macrophages are known to contribute to the innate immune response of the host by defending against pathogens infection, cancers, and immunological diseases (Hirayama et al., 2018). Macrophages are activated by mitogen‐activated protein kinases (MAPKs), nuclear factor‐kappa β (NF‐κβ), or phosphoinositide‐3‐kinase (PI3K)/protein kinase B (AKT) signaling pathway and secrete various immunomodulators once the invasion of the external harmful factors (Fang et al., 2017). Furthermore, many polysaccharides demonstrated that they opposed immunomodulatory activity in vitro and in vivo, by activating immune cells, regulating the expressions of pro‐inflammatory, and anti‐inflammatory cytokines in the lipopolysaccharide (LPS)‐stimulated RAW264.7 cells (Ramberg et al., 2010; Yin et al., 2019; Zhang et al., 2016). To our knowledge, little research carried out on *S. constricta* for its bioactive substances. Yuan and et al., (2020) extracted polysaccharides from *S*. *constricta* and evaluated its antioxidant activity. However, the information of the immunomodulatory activity of the polysaccharides from *S. constricta* still be unknown.

In the present study, a novel polysaccharide from *S. constricta* (SCP‐1‐1) was isolated and purified. Moreover, the immunomodulatory activity of SCP‐1‐1 was evaluated using the RAW 264.7 cell model in vitro. This study will enrich our understanding of the structure characteristics and bioactivities of the *S. constricta* polysaccharide and benefit further investigations into the utilization of similar mollusks.

## 2 MATERIALS AND METHODS

### 2.1 Materials and chemicals

*Sinonovacula constricta* was obtained from the local market (Ningbo, Zhejiang province) in March, 2019. All standard monosaccharides (glucose, mannose, galactose, arabinose, glucose, rhamnose, and xylose), DPPH, and ABTS were obtained from Sigma Chemical Co. (St. Louis MO, USA). Papain (100,000 U/g) was purchased from Shanghai Yuanye Biological Technology Co., Ltd. The other reagents were analytical grade and obtained from Sinopharm Chemical Reagent Co., Ltd.

### 2.2 Extraction and purification of polysaccharides from *Sinonovacula constricta* (SCPs)

*Sinonovacula constricta* was cleaned and separated the shell and the flesh. The flesh was cleaned and smashed with deionized water (1:2, w/v). The flesh homogenate was treated using papain and deionized water (1:10, g/ml) at 50°C for 3 h. After finishing hydrolysis, the hydrolysate was inactivated by heating at 100°C for 5 min and centrifuged at 2,150 × *g* for 10 min. The protein in the supernatants was removed by Sevag method. The supernatants were concentrated one quarter of the original volume of the solution. Absolute ethanol (3 volumes) added into the concentrated solution, then the mix solution was precipitated at 4°C for 24 h. The precipitate was collected by centrifugation at 1,210 × *g* for 10 min, following washed twice with absolute ethanol, dissolved in deionized water, and vacuum freeze‐dried to obtain a crude *S. constricta* polysaccharide (SCPs). The contents of SCPs were determined according to the phenol–sulfuric acid method (Dubois et al., 1956) with some modifications, and d‐glucose was used as the standard.

The crude SCPs sample (10 mg/ml, 10 ml) was separated and purified with a DEAE agarose gel‐FF column (2.6 cm × 30 cm). The elution process was carried out using deionized water and 1.0 M NaCl solution (1.0 ml/min). The main fractions were further purified by Sephacryl S‐400 HR (1.6 cm × 60 cm) column. The eluates were collected using an automated collector (BS‐100A, Shanghai, Huxi Analytical Instrument Co., Ltd.) and detected using the phenol–sulfuric acid method (Dubois et al., 1956; Yuan et al., 2020).

### 2.3 Structure characterization of SCPs

#### 2.3.1 Molecular weight determination

Molecular weight of SCPs was determined by high‐performance gel‐permeation chromatography (HPGPC) using a Waters Alliance 2695 HPLC (Milford, MA, USA) and column (300 mm × 7.8 mm; Ultrahydrogel™ Linear column, Waters corporation, Shanghai, China). The columns were calibrated with Glucose 180. T‐series Dextran (180, 2,700, 9,750, 36,800, 133,850, and 2,000,000 Da) were used as the reference compounds. A 20.0 μl aliquot of the sample (1 mg/ml) was injected for each run. Signals were processed online by GPC software package (Agilent Advance Bio SEC, USA) (Wang et al., 2018).

#### 2.3.2 Monosaccharide composition analysis

The monosaccharide composition of SCPs was analyzed using High‐Performance Liquid Chromatography (HPLC). SCPs was firstly hydrolyzed with trifluoroacetic acid at 110°C for 8 h. Then, the monosaccharides of SCP‐1‐1 were converted into their corresponding acetylated derivatives with PMPs, PMP derivatives were eluted (1 ml/min) by thermo hypersil ODS‐2 HPLC columns (250 mm × 4.6 mm) at 25°C. The absorbance rate was monitored at the wavelength of 245 nm (Liu et al., 2019). Seven monosaccharides, including glucose, mannose, galactose, arabinose, rhamnose, and xylose were chosen as the standards.

#### 2.3.3 Fourier transform infrared spectroscopy (FT‐IR) analysis

The samples were thoroughly ground with the air‐dried KBr (100 mg) powder and pressed into pellets. Fourier transform infrared spectroscopy (FT‐IR) spectra were acquired (resolution, 4 cm^−1^) on a Frontier spectrophotometer (PerkinElmer, Shelton, CT, USA) in the vibration range of 400–4,000 cm^−1^ for three times (Qin et al., 2018).

#### 2.3.4 Nuclear magnetic resonance (NMR) analysis

The samples (20 mg) were treated with 99.98% D_2_O (Sigma‐Aldrich, Shanghai, China) three times and lyophilized. One‐ and two‐dimensional NMR spectra were recorded at 298 K using Bruker AVANCEIII 600 spectrometer with cryoprobe (AV‐500 MHz spectrometer, Bruker, Rheinstetten, Germany). The obtained proton and carbon shifts were expressed as parts per million (ppm) (Rajasekar et al., 2019; Zhang et al., 2021).

### 2.4 Immunomodulatory activity assay

#### 2.4.1 Cell line and culture

RAW 264.7 cells were obtained from the Shanghai Cell Bank of Chinese Academy of Sciences (Shanghai, China) and pre‐cultured in an DMEM medium supplemented with 10% (v/v) fetal bovine serum, penicillin–streptomycin solution (100 μg/ml) in a humidified incubator with 5% CO_2_ at 37°C. RAW 264.7 cells were cultured and harvested at the logarithmic growth phase.

#### 2.4.2 Assessment of cell proliferation

The cell viability was determined by CCK‐8 method (Li et al., 2014). RAW 264.7 cells (1.0 × 10^5^ cells/ml, 100 μl) were incubated in a 96‐well plate at 37°C for 12 h in a humidified incubator with 5% CO_2_. The non‐adherent cells were removed by washing with PBS (0.1 M, pH 7.2). Then, control and different concentrations of SCPs (100 μl/well) were added and incubated for 24 h. The DMEM medium without SCPs was chosen as the control group (blank), PBS as the control group (10 μg/ml, negative), LPS as the control group (2 μg/ml, positive), and different concentrations of SCPs, respectively. At the end of culture, the cells were washed twice with PBS, and then 10‐μl CCK‐8 solution (5 mg/ml in the DMEM medium) was added. The plate was further incubated at 37°C for 4 h in a humidified incubator with 5% CO_2_. After the untransformed CCK‐8 was removed by pipetting, 150‐μl DMSO solution/well was added and incubated for 10 min. Cell viability was calculated by the following equation:

$$\text{Cell}\;\text{viability}\left(\%\right)=\frac{{\text{Abs}}_{\left(\text{sample}\right)}}{{\text{Abs}}_{\left(\text{Blank}\;\text{control}\right)}}\times 100$$

#### 2.4.3 Assay of phagocytosis

The phagocytosis of RAW 264.7 cells was determined by the neutral red staining method (Wang et al., 2017). The cell suspension (1.0 × 10^5^ cells/ml in DMEM) was seeded into a 96‐well plate (100 μl/well) and allowed in a humidified incubator with 5% CO_2_ to adhere at 37°C for 24 h. The non‐adherent cells were removed by washing twice with PBS (0.1 M, pH 7.2). Then, different concentrations of SCPs (300, 500, 750, and 1,000 μg/ml, respectively) were added followed by incubation for another 48 h. LPS (2 μg/ml) and the DMEM medium in the absence of polysaccharide were used as a positive control and a blank control, respectively. At the end of incubation, 20 μl of 0.1% (w/v) neutral red solution (in normal saline) was added and incubated in a humidified incubator with 5% CO_2_ at 37°C for 4 h. Following the supernatant was discarded and the cells were washed with PBS twice to remove excess neutral red. The cell lysate (200 μl/well) was added and kept for 10 min. Phagocytosis index was calculated by the following equation:

$$\text{Phagocytosis}\;\text{index}=\frac{{\text{Stomach muscles}}_{\left(\text{sample}\right)}}{{\text{Abs}}_{\left(\text{Blank}\;\text{control}\right)}}\times 100\%$$

#### 2.4.4 Determination of Nitric oxide (NO), TNF‐α, IL‐6, and IL‐1β production

NO released into the culture supernatant of RAW 264.7 cells was quantified by measuring the nitrite content. The total NO content was measured by the Griess method (Zhang et al., 2019). The cell suspension (1.0 × 10^5^ cells/ml) was seeded into a 96‐well plate (100 μl/well) and incubated at 37 ℃ for 24 h in a humidified incubator with 5% CO_2_. The non‐adherent cells were removed by washing twice with PBS buffer (0.1 M, pH 7.2). Then, SCPs (100 μl/well) at different concentrations (300, 500, 750, and 1,000 μg/ml) were, respectively, added into each well followed by incubation for another 24 h. Following, the supernatant of each well was collected for analysis of NO released by RAW 264.7 cells. Nitrite concentration was calculated from the NaNO_2_ standard curve (1, 2, 5, 10, 40, 60, and 100 μM, respectively) (Sun, Liu, et al., 2018; Sun, Gong, et al., 2018; Zha et al., 2015). The levels of tumor necrosis factor alpha (TNF‐α), interleukin 6 (IL‐6), and interleukin 1β (IL‐1β) were assayed using ELISA kits (Abcam, China) according to the manufacturer's instructions.

#### 2.4.5 Western blot analysis

RAW 264.7 cells were treated with different concentrations of SCPs solution (300, 500, 750, and 1,000 μg/ml) for 48 h. The cell lysate was extracted on ice and centrifuged at 13,980 × *g* at 4°C for 20 min. The concentration of total protein was quantitated using a BCA protein assay kit (Beyotime, Shanghai, China). The cytosolic proteins were denatured by boiling in a loading buffer for denaturation. Equal amounts of protein (1 μg/μl) were loaded on the 10% SDS‐PAGE gel and transferred onto a 0.45‐μm polyvinylidene difluoride (PVDF) membrane (GE Healthcare, USA). Subsequently, the membrane was blocked using 5% non‐fat milk in TBST ((0.1% (v/v) Tween 20, 20 mM Tris‐HCl, 150 mM NaCl) for 1 h at 25°C, following incubation at 4°C overnight with the primary antibodies. These membranes were then washed three times (5 min/time) with TBST and incubated with the corresponding secondary antibody at 25°C for 1 h (Liu et al., 2019; Rong et al., 2019). Immune complexes were visualized by a detection system using an enhanced chemiluminescence kit (Bio‐Rad).

### 2.5 Statistical analysis

All experiments were independently repeated at least three times. The data were presented as means ± standard deviation (*SD*). One‐way analysis of variance (ANOVA) with the Duncan's multiple range tests was used for statistical analysis. *p* < .05 was considered as statistically significant.

## 3 RESULT AND DISCUSSION

### 3.1 Isolation and purification of SCPs

**FIGURE 1:**
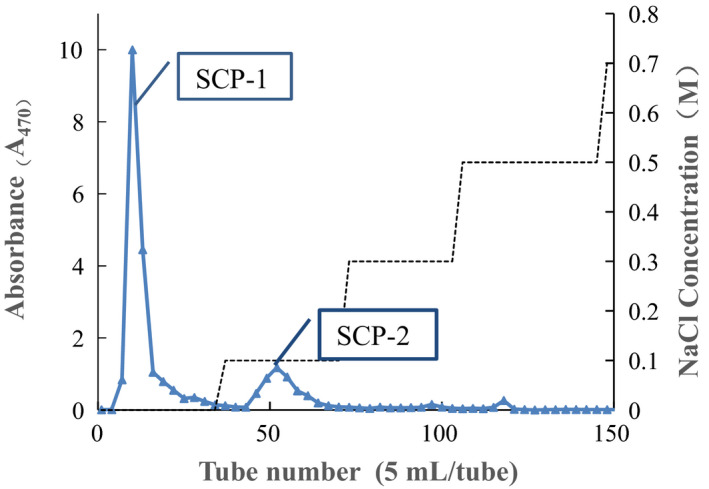
DEAE‐Sepharose‐FF weak anion exchange chromatography of SCPs

**FIGURE 2:**
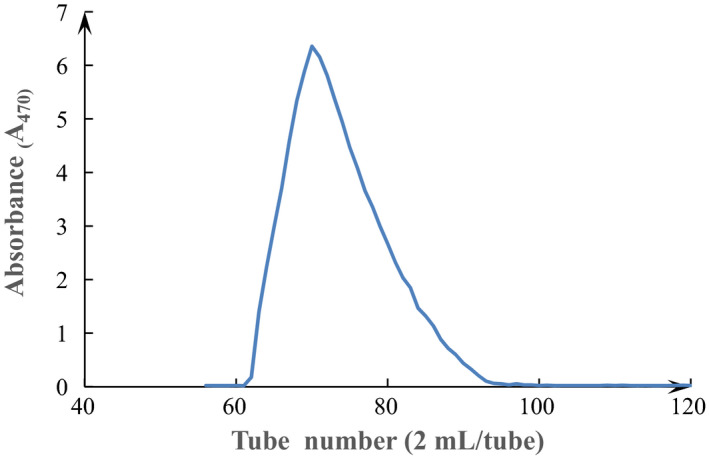
Sephacryl S‐400 HR chromatography of SCP‐1‐1

The yield of crude SCPs was about 0.62% (w/w), but the carbohydrate content of in the crude SCPs by the phenol–sulfuric acid method reached 81.3% (w/w). The crude SCPs by enzymolysis extraction was fractioned by DEAE Sepharose‐FF column. The eluent curve of the SCPs is shown in Figure 1. The peaks of two polysaccharide fraction were separated and collected, respectively. Based on their immunomodulatory activity, SCP‐1 was considered over the other fractions and subjected to subsequent investigations. SCP‐2 was not further purified because of its lower immunomodulatory activity than that of SCP‐1. Then, SCP‐1 fraction was further purified using Sephacryl S‐400 HR and gained one main sub‐fractions (SCP‐1‐1) (Figure 2). SCP‐1‐1 was collected and further analyzed.

### 3.2 Structure characteristics of SCPs

#### 3.2.1 Molecular weight and monosaccharide composition analysis

As high‐performance gel‐permeation chromatography (HPGPC) is considered a reliable method for measuring the homogeneity of polysaccharides (Xie et al., 2015). The results showed that SCP‐1‐1 fraction was only a single symmetrical peak and a highly homogeneous polysaccharide with an average molecular weight of about 440.5 kDa. SCP‐1‐1 was mainly composed of glucose and mannose. The molecular weight and monosaccharide composition of SCP‐1‐1 in this study are different from that described by Yuan et al. (2020), which may be due to the difference of the raw material (location, season, etc.), enzyme, and separation protocols.

#### 3.2.2 Fourier transform infrared spectroscopy (FT‐IR) analysis

**FIGURE 3:**
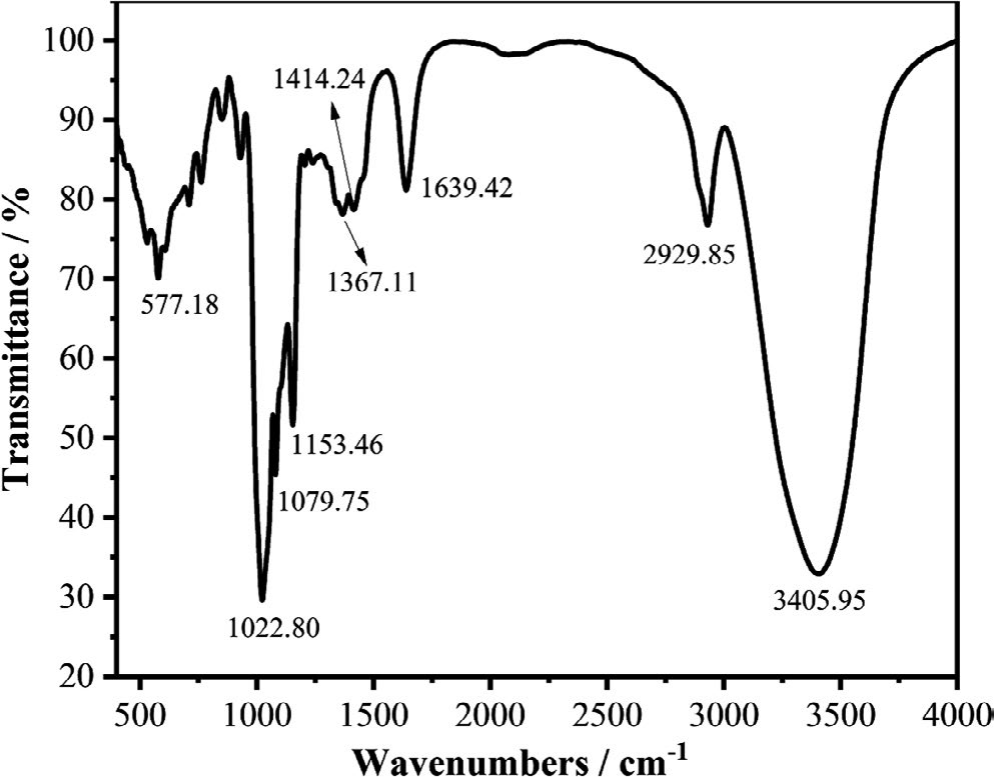
FT‐IR spectra of SCP‐1‐1 in the range of 4,000–400 cm^−1^

For the samples, a strong and wide peak at approximately 3,406 cm^−1^ corresponding to hydrogen‐bonded hydroxyl group. An intense peak at 2,930 cm^−1^ was assigned to C‐H stretching vibration. These peaks at 1,639 and 1,412 cm^−1^ were attributed to C=O stretching vibration. The bands extended from 1,485 cm^−1^ to 1,350 cm^−1^ were assigned to ‐CH (O‐CH_2_) flexural vibrations. The absorption bands between 1,200 and 1,020 cm^−1^ were due to C‐O (C‐O‐H, C‐O‐C) stretching vibrations. The peak at 930 cm^−1^ was caused by the C‐O (O‐CH_2_) stretching vibration of glycosidic bonds. The characteristic absorption band at 850 cm^−1^ indicated the presence of α‐type glycosidic bond in the SCP‐1‐1 (Dong et al., 2020a; Huang et al., 2020). In conclusion, the classical absorption bands in the FT‐IR (Figure 3) reflect the carbohydrate nature of SCP‐1‐1.

#### 3.2.3 Nuclear magnetic resonance (NMR) analysis

**FIGURE 4:**
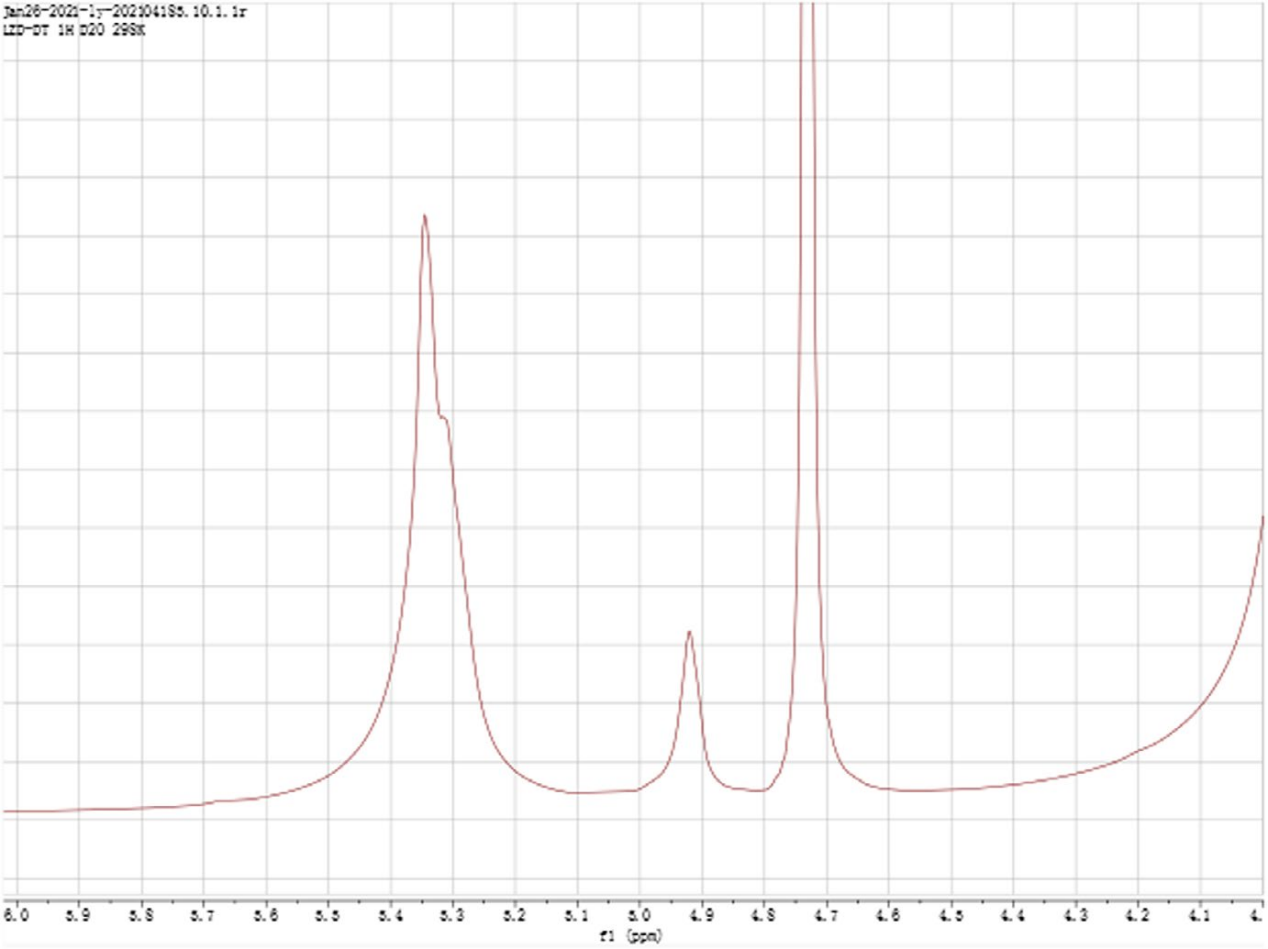
NMR ^1^H‐spectrum of SCP‐1‐1

**FIGURE 5:**
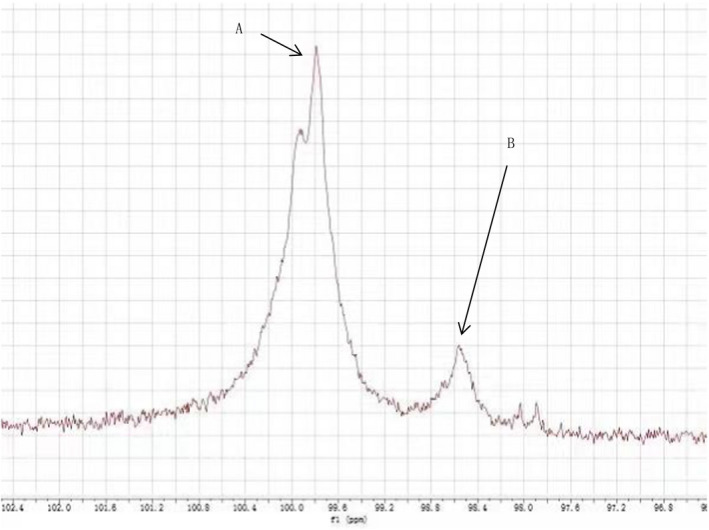
NMR ^13^C‐spectrum of SCP‐1‐1

NMR spectra are used for further elucidating the structure characterizations of SCP‐1‐1, including monosaccharide composition, anomeric configuration, and the types and sequences of the glycosidic linkages. Signals within the range of 3.5–5.5 ppm (1H spectra) and 97–102 ppm (13C spectra) showed a typical characteristic of polysaccharide, respectively (Sun et al., 2019). Signals of hydrogen protons on α‐configuration glycoside anomeric carbon generally occurred in the range of δ 5.0–5.8 ppm in 1H NMR spectra, while the isomers of β‐configuration glycosides occurred in the range of δ 4.4–5.0 ppm 1H NMR spectra (Song et al., 2020). As shown in Figure 4, the chemical shifts at δ 5.32 and 4.95 ppm were attributed to two anomeric protons. These results indicated the existence at least one α‐configuration and two β‐configurations in the glycosidic bond of SCP‐1‐1, in which β‐constitution was dominant. As shown in Figure 5, two main signal peaks (δ 99.805 and δ 98.554 ppm) appeared in the isomeric carbon region (δ 95–110 ppm), indicating that there were two monosaccharides in SCP‐1‐1, which were consistent with the monosaccharide composition analysis of LNP‐1 (Shu et al., 2019).

### 3.3 Immunomodulatory activity assay

#### 3.3.1 Assessment of cell proliferation

**FIGURE 6:**
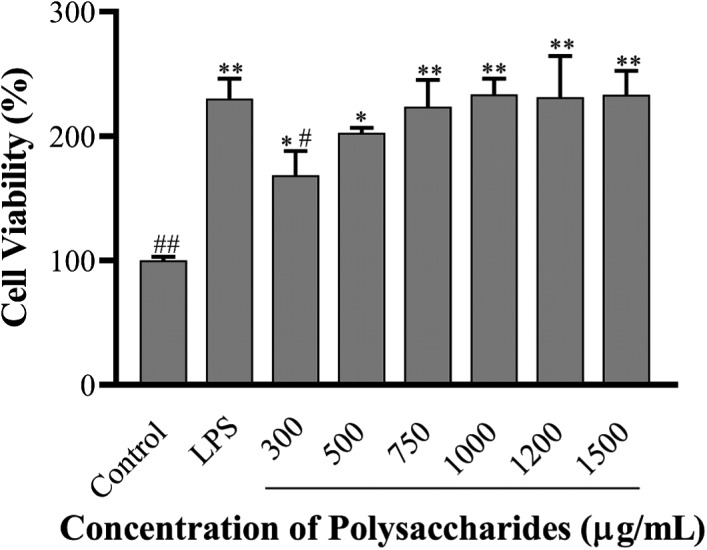
Effect of SCP‐1‐1 on the proliferation of RAW264.7 cell. *p < .05 or **p < .01 versus Control; ^#^ *p* < .05 or ^##^ *p* < .01 versus LPS

LPS‐induced RAW 264.7 cell (mouse macrophages cell lines) is commonly employed as the anti‐inflammation model for screening anti‐inflammation candidate in vitro. As shown in Figure 6, the cell viability showed an increase in a dose‐dependent manner (*p* < .05) at 300–1,000 μg/ml concentrations of SCP‐1‐1 compared with the control group. The cell viability of SCP‐1‐1 groups increased compared with LPS group; however, there was no significant difference (*p* > .05). The viability of RAW264.7 cell treated with 1,000 μg/ml SCP‐1‐1 for 24 hr was 285.87% reaching to the maximum. A similar result has been reported of the polysaccharide from *Cassia obtusifolia*, with the proliferation rate higher than its two sub‐fractions CP‐30 (30% ethanol precipitate) and CP‐40 (40% ethanol precipitate) (Feng et al., 2016). These results indicated that SCP‐1‐1 had no cytotoxic effect on RAW264.7 cells within a certain concentration range (300–1,500 μg/ml).

#### 3.3.2 Assay of phagocytosis

**FIGURE 7:**
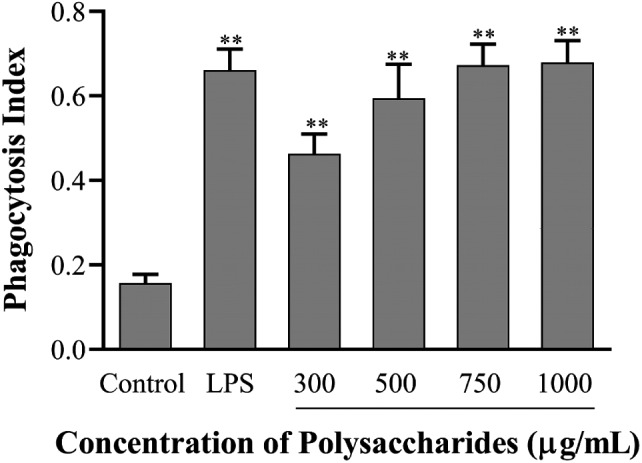
Effect of SCP‐1‐1 on the phagocytosis of RAW264.7 cell. *p < .05 or **p < .01 versus Control; ^#^ *p* < .05 or ^##^ *p* < .01 versus LPS

**FIGURE 8:**
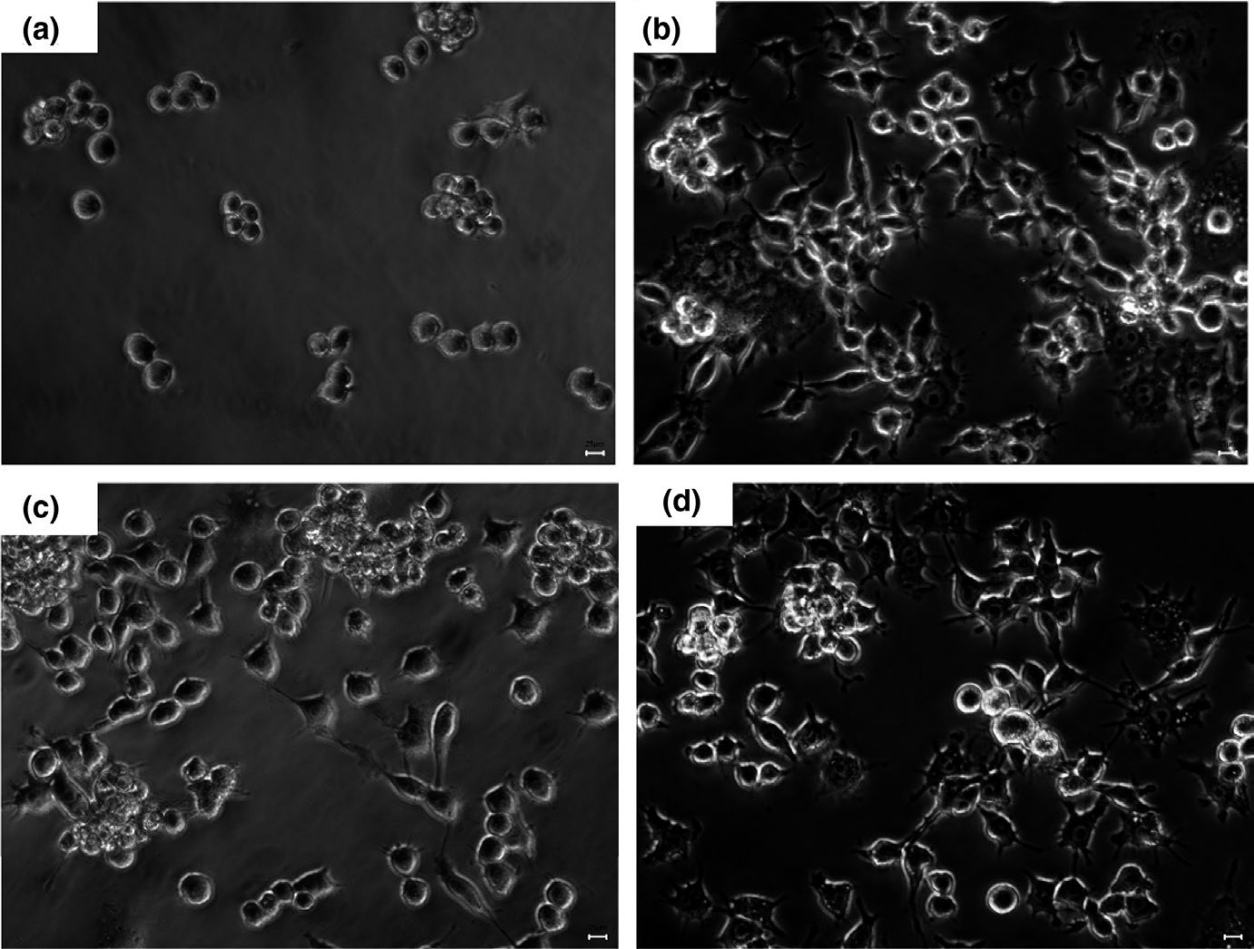
Effect of SCP‐1‐1 on the morphology of RAW264.7 cell (400×). (a) Control; (b) LPS; (c) 300 μg/ml SCP‐1‐1; (d) 1,000 μg/ml SCP‐1‐1

Phagocytosis is an important defense mechanism against pathogens invasion and dead or expired blood and tissue cells in vertebrates (Dong et al., 2020b). The increase of phagocytosis is the primary and distinguishing feature of macrophage activation (Gordon, 2016). As shown in Figure 7, the phagocytic capacity increased with the increasing of the concentrations of SCP‐1‐1. The phagocytosis of RAW264.7 cell in SCP‐1‐1 groups significantly increased (*p* < .05) compared with the control group. However, the stimulatory effect of SCP‐1‐1 (1,000 μg/ml) on the phagocytic rate of macrophages was similar with the LPS group and stronger than that of the control group. The images of fluorescence microscope showed that the fluorescence intensity depending on the macrophages treated with SCP‐1‐1 or LPS was obviously stronger than that in the control group (Figure 8). These results indicated that SCP‐1‐1 could effectively enhance its immunomodulatory effect through moderately promoting the phagocytic activities of macrophages. Wang et al., (2021) also reported that polysaccharide fractions from asparagus (*Asparagus officinalis* L.) skin had higher immunomodulatory activity.

#### 3.3.3 Determination of NO, TNF‐α, IL‐6, and IL‐1β

**FIGURE 9:**
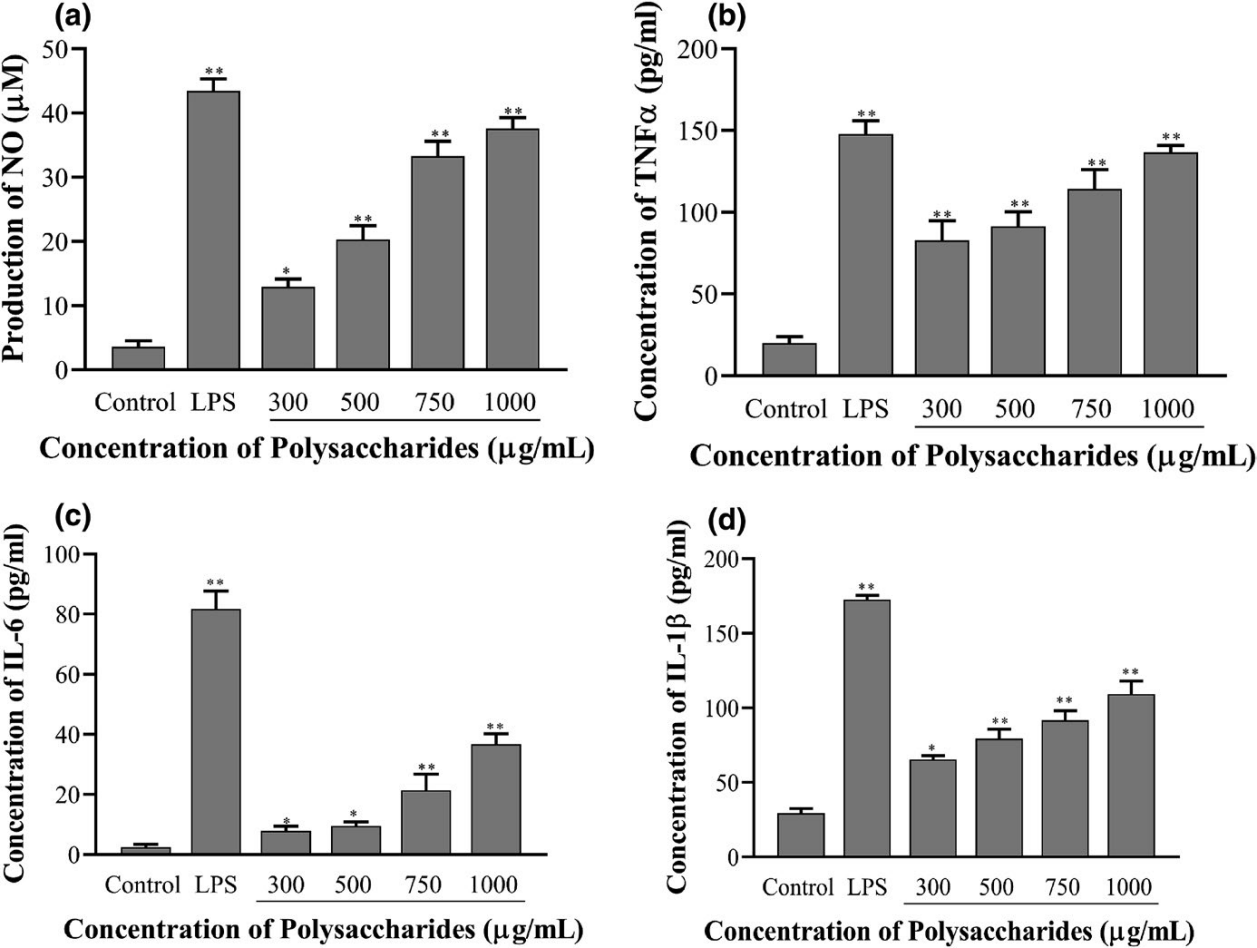
Effect of SCP‐1‐1 on RAW264.7 cell‐induced NO production. *p < .05 or **p < .01 versus Control

NO is one of the signaling molecules related to macrophage cytolytic function, and is crucial for fighting against microbes, parasites, and tumor cells (Zhang et al., 2020). Cytokines are produced by macrophages and lymphocytes mediate the unleashing of the effective immune response, link the innate and adaptive immunity to induce the necrosis, apoptosis, and acute inflammation responsible for some intracellular signaling events (Huynh et al., 2007). Macrophage activation has become one of the important indicators for improving the body's innate immune system (Hirayama et al., 2018). Thus, macrophage activation is also an important symbol for evaluating on whether polysaccharides have immunomodulatory function. Once macrophages are activated, the secretion of NO and some cytokines (TNF‐α, IL‐6, and IL‐1β) are increased. RAW264.7 cells can spontaneously secrete cytokines in the resting state, and such secretion in RAW264.7 cells was significantly promoted by LPS stimulation. The stimulation of LPS simultaneously promoted the production of NO, TNF‐α, IL‐6, and IL‐1β of RAW 264.7 cells. The secretion of NO was significantly increased by SCP‐1‐1 with a dose dependently (300–1,000 μg/ml). In addition, RAW 264.7 cells treated with SCP‐1‐1 at various concentrations (300, 500, 750, and 1,000 μg/ml) showed significant production of NO (12.95 μM, 20.31 μM, 33.27 μM, and 37.61 μM, respectively), increased by 3.59, 5.64, 9.24, and 10.45 folds compared to the control group (3.60 μM), respectively. The levels of TNF‐α production in RAW 264.7 cells treated with SCP‐1‐1 at concentration of 300, 500, 200, 750, and 1,000 μg/ml were increased by 4.17, 4.61, 5.76, and 6.88 folds, respectively (Figure 9b) compared with the control group. The contents of TNF‐α in the SCP‐1‐1 treated groups increased significantly (*p* < .01) compared with the LPS group. The contents of SCP‐1‐1 treated group increased; however, there was no significant difference (*p* > .05). Furthermore, the levels of IL‐6 production in RAW 264.7 cells treated with SCP‐1‐1 at concentration of 300, 500, 200, 750, and 1,000 μg/ml were also increased significantly by 3.24, 3.92, 8.79, and 15.09 folds, respectively (Figure 9c). The content of IL‐6 in SCP‐1‐1 groups increased significantly (*p* < .05) compared with control group. Similarly, the levels of IL‐1β production in RAW 264.7 cells treated with SCP‐1‐1 group at various concentrations were also increased significantly by 2.23, 4.61, 5.76, and 6.89 folds, respectively (Figure 9d). The content of IL‐1β in SCP‐1‐1 groups increased significantly (*p* < .05) compared with control group. This result was in accordance with the study of Huang et al. (2021), who reported the immunomodulatory activity of pectic polysaccharide from *Cucurbita moschata* Duch. The result indicated that SCP‐1‐1 could significantly enhance the macrophages' activities by inducing the production of NO, TNF‐α, IL‐6, and IL‐1β with a dose dependently.

#### 3.3.4 Western blot analysis

**FIGURE 10:**
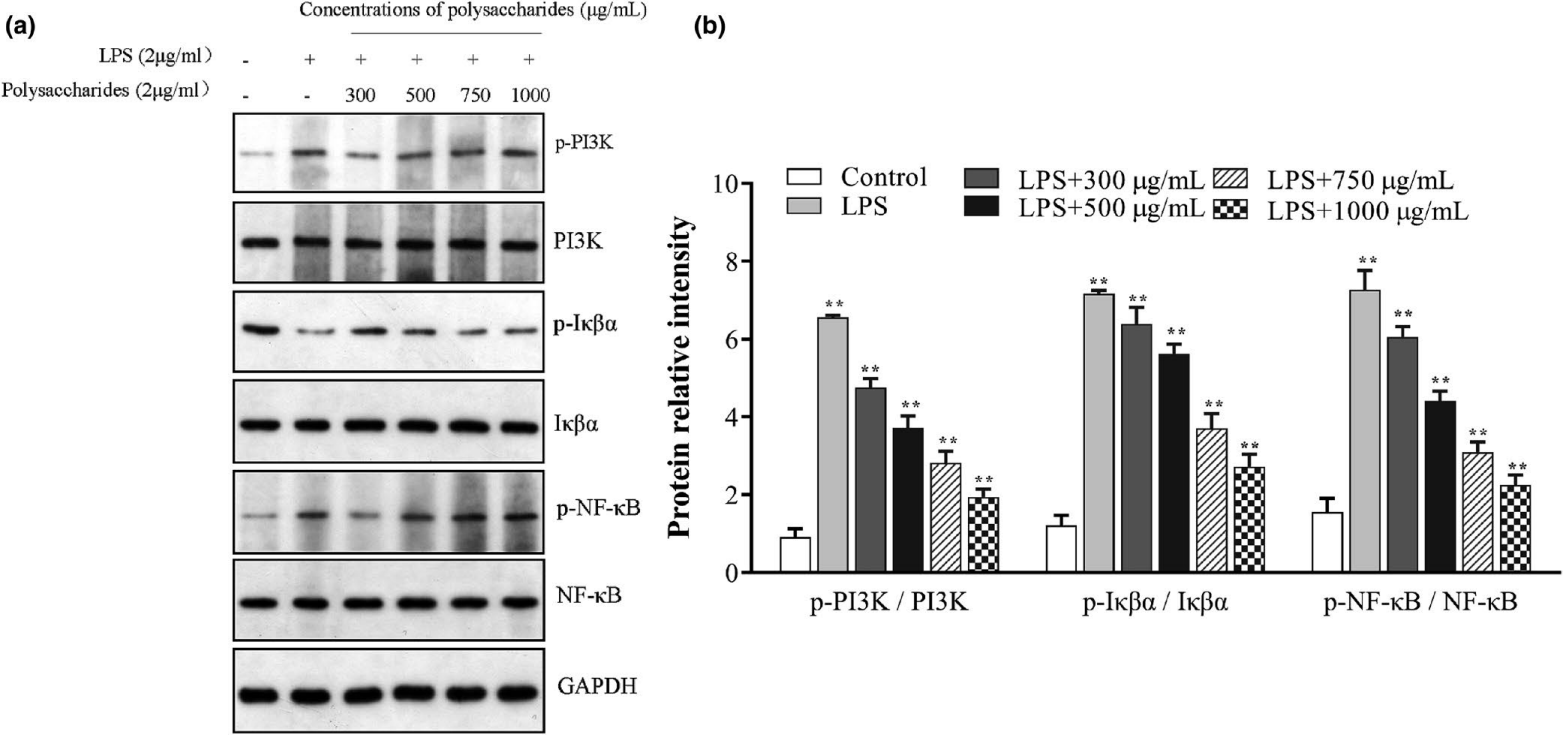
Effect of SCP‐1‐1 on the signaling pathways of RAW264.7 cell by Western blot assay. *p < .05 or **p < .01 versus Control; ^#^ *p* < .05 or ^##^ *p* < .01 versus LPS

NF‐κβ plays an important role in the immune modulation and inflammatory responses (Ren et al., 2017). Activated NF‐κβ can promote the secretion of cytokines (TNF‐α, IL‐1β, etc). Thus, the effects of pro‐inflammatory mediators (such as NF‐κβ) blocked could be considered an effective therapeutic strategy (Janeway & Medzhitov, 2002). The activation of the NF‐κβ signaling pathway by TLR2 (toll‐like receptor 2)/TLR4 (toll‐like receptor 4) began as IKK activated by TLR2/TLR4 phosphorylates Iκβ‐α and subsequently Iκβ‐α was degraded (Kawai & Akira, 2017). Western blotting experiments were performed to assay the levels of key proteins in the signaling pathways of RAW264.7 cells to gain further insights on how SCP‐1‐1 inhibits the growth of cancer cells and promotes apoptosis (Yuan et al., 2013). PI3K‐Akt‐NF‐κβ plays a role in anti‐inflammatory function. The level of phosphatidylinositide 3‐kinase (PI3K), p‐PI3K, Iκβα, p‐Iκβα, NF‐κβ, and p‐NF‐κβ regulating apoptosis in the mitochondrial apoptotic pathway were examined. The expression level of p‐PI3K and p‐NFκβ in the SCP‐1‐1 group increased significantly in a concentration‐dependent manner (*p* < .05) compared with the control group. When the RAW264.7 cells treated by LPS, the expression level of p‐PI3K, p‐Iκβα, and p‐NF‐κβ was significantly down‐regulated (*p* < .01). The SCP‐1‐1 treated group was significantly darker, which suggested that the protein expression of p‐PI3K, p‐Iκβα, and p‐NF‐κβ was significantly expressed in LPS‐stimulated RAW264.7 cells (Figure 10). SCP‐1‐1 could induce the phosphorylation of PI3K and Iκβ‐α and the degradation of Iκβ‐α in RAW264.7 cells. Similarly, the protein bands were gradually shallower and thinner with the SCP‐1‐1 treated group in a dose‐dependent manner (*p* < .01). Moreover, the inhibitory effects are becoming increasingly clear with the increasing of SCP‐1‐1 concentrations. Liu et al. reported the immunomodulatory activity of the polysaccharides from the lignified okra that gained similar results (Liu et al., 2021). Xie et al., pointed out that the polysaccharides from *Phellinus linteus* played a key role in preventing the translocation of NF‐κβ and the phosphorylation of its inhibitor Iκβ (Xie et al., 2019). Based on these results, it is thought that SCP‐1‐1 may activate RAW264.7 cell by NF‐κβ and PI3K signaling pathway in macrophage.

## 4 CONCLUSION

A polysaccharide from *Sinonovacula constricta* (SCP‐1‐1) was isolated, purified, and characterized. SCP‐1‐1 mainly consisted of glucose and mannose with the molecular weight of 440.0 kDa. The immunomodulatory activity results demonstrated that the immunomodulatory activity of SCP‐1‐1 on RAW264.7 cells was activated by enhancing phagocytic ability of macrophage and promoting NO production and some cytokines (TNF‐α, IL‐6, and IL‐1β) secretion. Furthermore, western blot results showed that SCP‐1‐1 could regulate the expression levels of the key proteins in NF‐κβ and PI3K signaling pathways. These results showed that SCP‐1‐1 could serve as an honest candidate for immunomodulatory purpose for a therapeutical agent or an ingredient of functional foods. The molecular structure of SCP‐1‐1 and its immunomodulatory activity using animal evaluation models in vivo in the future will be focused because this research is a cell‐based study.

## CONFLICT OF INTEREST

The authors declare that they have no conflict of interest.

## ETHICAL STATEMENT

This study does not involve any human or animal testing.

## Data Availability

The data used to support the findings of this study are available from the corresponding author upon reasonable request.
